# Risk of Zika microcephaly correlates with features of maternal antibodies

**DOI:** 10.1084/jem.20191061

**Published:** 2019-08-14

**Authors:** Davide F. Robbiani, Priscilla C. Olsen, Federico Costa, Qiao Wang, Thiago Y. Oliveira, Nivison Nery, Adeolu Aromolaran, Mateus S. do Rosário, Gielson A. Sacramento, Jaqueline S. Cruz, Ricardo Khouri, Elsio A. Wunder, Adriana Mattos, Bruno de Paula Freitas, Manoel Sarno, Gracinda Archanjo, Dina Daltro, Gustavo B.S. Carvalho, Kleber Pimentel, Isadora C. de Siqueira, João R.M. de Almeida, Daniele F. Henriques, Juliana A. Lima, Pedro F.C. Vasconcelos, Dennis Schaefer-Babajew, Stephanie A. Azzopardi, Leonia Bozzacco, Anna Gazumyan, Rubens Belfort, Ana P. Alcântara, Gustavo Carvalho, Licia Moreira, Katiaci Araujo, Mitermayer G. Reis, Rebekah I. Keesler, Lark L. Coffey, Jennifer Tisoncik-Go, Michael Gale, Lakshmi Rajagopal, Kristina M. Adams Waldorf, Dawn M. Dudley, Heather A. Simmons, Andres Mejia, David H. O’Connor, Rosemary J. Steinbach, Nicole Haese, Jessica Smith, Anne Lewis, Lois Colgin, Victoria Roberts, Antonio Frias, Meredith Kelleher, Alec Hirsch, Daniel N. Streblow, Charles M. Rice, Margaret R. MacDonald, Antonio R.P. de Almeida, Koen K.A. Van Rompay, Albert I. Ko, Michel C. Nussenzweig

**Affiliations:** 1 Laboratory of Molecular Immunology, The Rockefeller University, New York, NY; 2 Laboratory of Virology and Infectious Disease, The Rockefeller University, New York, NY; 3 Howard Hughes Medical Institute, The Rockefeller University, New York, NY; 4 Faculdade de Farmácia, Universidade Federal do Rio de Janeiro, Rio de Janeiro, Brazil; 5 Key Laboratory of Medical Molecular Virology (MOE/NHC/CAMS), School of Basic Medical Sciences, Shanghai Medical College, Fudan University, Shanghai, China; 6 Instituto Gonçalo Moniz, Fundação Oswaldo Cruz/MS, Salvador, Bahia, Brazil; 7 Department of Epidemiology of Microbial Diseases, Yale School of Public Health, New Haven, CT; 8 Faculdade de Medicina and Instituto da Saúde Coletiva, Universidade Federal da Bahia, Salvador, Bahia, Brazil; 9 Hospital Geral Roberto Santos, Secretária da Saúde do Estado da Bahia, Salvador, Brazil; 10 Instituto Evandro Chagas, Ministério da Saúde Ananindeua, Pará, Brazil; 11 Universidade Federal de São Paulo, São Paulo, Brazil; 12 Hospital Aliança, Salvador, Bahia, Brazil; 13 Hospital Santo Amaro, Salvador, Bahia, Brazil; 14 California National Primate Research Center, University of California, Davis, Davis, CA; 15 Department of Pathology, Microbiology, and Immunology, School of Veterinary Medicine, University of California, Davis, Davis, CA; 16 Washington National Primate Research Center, Seattle, WA; 17 Center for Innate Immunity and Immune Disease, University of Washington, Seattle, WA; 18 Department of Immunology, University of Washington, Seattle, WA; 19 Department of Global Health, University of Washington, Seattle, WA; 20 Department of Pediatrics, University of Washington, Seattle, WA; 21 Department of Obstetrics and Gynecology, University of Washington, Seattle, WA; 22 Center for Global Infectious Disease Research, Seattle Children’s Research Institute, Seattle, WA; 23 Department of Pathology and Laboratory Medicine, University of Wisconsin-Madison, Madison, WI; 24 Wisconsin National Primate Research Center, University of Wisconsin-Madison, Madison, WI; 25 Division of Reproductive and Developmental Sciences, Oregon National Primate Research Center, Beaverton, OR; 26 Division of Pathobiology and Immunology, Oregon National Primate Research Center, Beaverton, OR; 27 Pathology Services Unit, Division of Comparative Medicine, Oregon National Primate Research Center, Beaverton, OR; 28 Vaccine and Gene Therapy Institute, Oregon Health and Science University, Portland, OR; 29 Department of Obstetrics and Gynecology, Oregon Health and Science University, Portland, OR

## Abstract

Zika virus (ZIKV) infection during pregnancy causes congenital abnormalities, including microcephaly. However, rates vary widely, and the contributing risk factors remain unclear. We examined the serum antibody response to ZIKV and other flaviviruses in Brazilian women giving birth during the 2015–2016 outbreak. Infected pregnancies with intermediate or higher ZIKV antibody enhancement titers were at increased risk to give birth to microcephalic infants compared with those with lower titers (P < 0.0001). Similarly, analysis of ZIKV-infected pregnant macaques revealed that fetal brain damage was more frequent in mothers with higher enhancement titers. Thus, features of the maternal antibodies are associated with and may contribute to the genesis of ZIKV-associated microcephaly.

## Abstract (precis)

ZIKV infection during pregnancy causes microcephaly, but not all neonates are affected. This study shows that features of the maternal antibodies correlate with the risk of ZIKV-associated microcephaly.

## Introduction

For decades, infection by Zika virus (ZIKV) went either unrecognized or occurred only sporadically and was associated with mild symptoms. ZIKV was detected in Brazil in 2015 and spread rapidly, reaching infection rates exceeding 60% (Zanluca et al., 2015; Netto et al., 2017; Rodriguez-Barraquer et al., 2019). During the Brazilian ZIKV outbreak, it was recognized that congenital infection can cause fetal abnormalities, including visual and hearing impairment, skeletal deformities, and microcephaly, with an overall prevalence of microcephaly estimated at 2.7 to 5.8% of live births from ZIKV-infected pregnancies and with a global rate of adverse outcomes exceeding 40% in some regions (Brasil et al., 2016; Kleber de Oliveira et al., 2016; Rasmussen et al., 2016; Coelho and Crovella, 2017; Hoen et al., 2018). Why some ZIKV-infected pregnant women deliver apparently healthy newborns while others have babies with microcephaly is unknown.

Pre-existing antibodies to dengue virus (DENV) have the potential to augment the risk of severe dengue disease upon infection with a new DENV serotype, and the increased hazard relates directly to pre-exposure DENV antibody titers (Katzelnick et al., 2017; Salje et al., 2018). Since ZIKV and DENV are antigenically related flaviviruses, it was suggested that prior DENV exposure may also influence vertical ZIKV transmission (Andrade and Harris, 2017; Halstead, 2017; Miner and Diamond, 2017). According to this hypothesis, antibodies to DENV that are cross-reactive with, but fail to neutralize, ZIKV might worsen fetal disease (Dejnirattisai et al., 2016; Priyamvada et al., 2016; Bardina et al., 2017). In mouse models and ex vivo human placental cultures, such antibodies can contribute to increased infection and pathology (Zimmerman et al., 2018; Brown et al., 2019; Rathore et al., 2019). Although there is no evidence that anti-DENV antibodies predispose humans to abnormal birth outcomes (Halai et al., 2017; Moreira-Soto et al., 2017; Pedroso et al., 2019), the potential association of maternal antibodies that enhance ZIKV infection in vitro with microcephaly, the most severe outcome of congenital Zika syndrome (CZS), has not been evaluated (Andrade and Harris, 2017; Halstead, 2017; Miner and Diamond, 2017).

Here, we report on the maternal serologic correlates of ZIKV-associated microcephaly and brain injury in both human neonates during the Brazilian ZIKV outbreak of 2015–2016 and in experimental ZIKV infection of pregnant macaques.

## Results

### Increased in vitro ZIKV neutralizing capacity in mothers of microcephalic newborns

**Figure 1.**
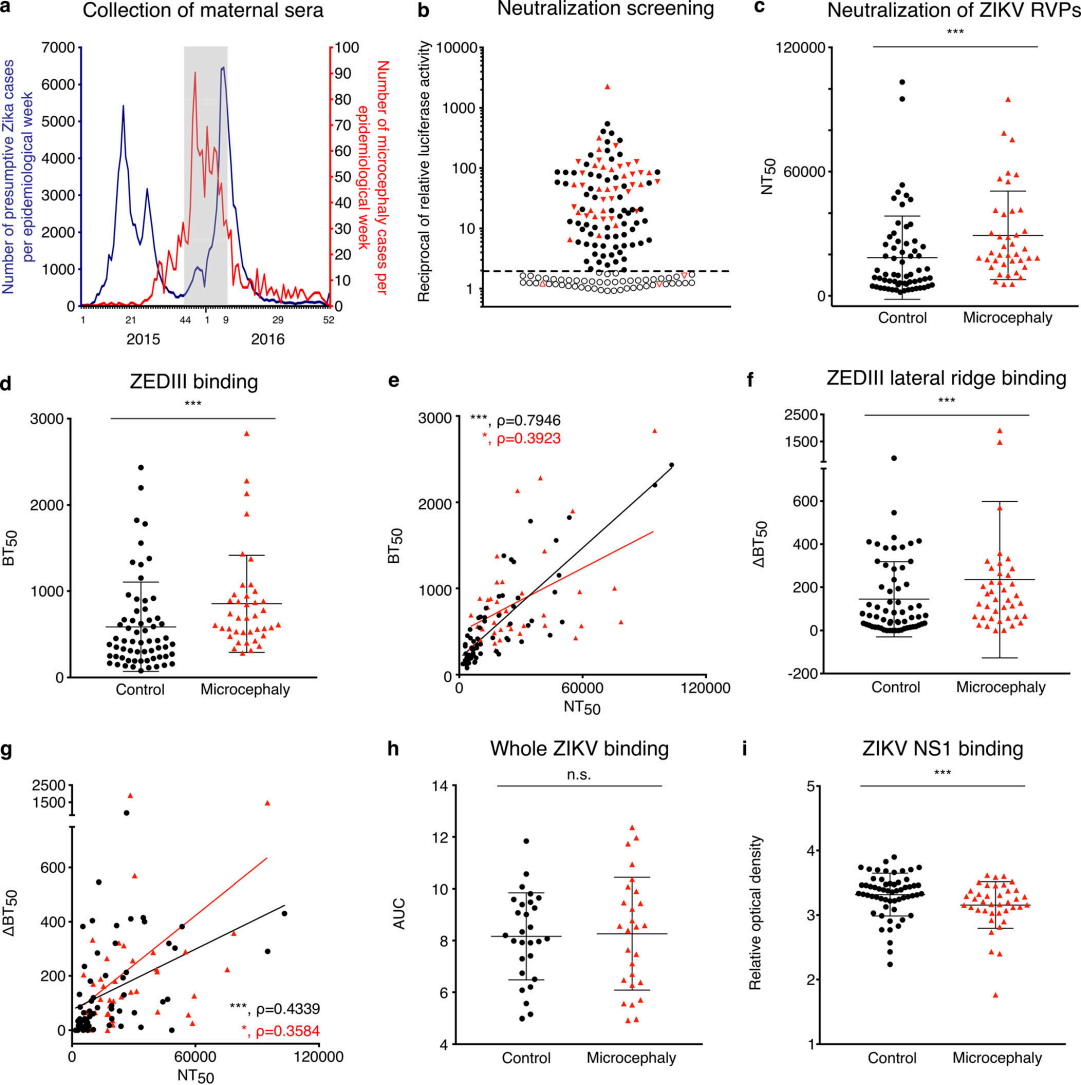
**ZIKV neutralization capacity and ZEDIII binding are increased in mothers of microcephalic newborns. (a)** The collection period of the maternal sera of this study (November 2015 to February 2016) is indicated in gray alongside the incidence of exanthematic diseases (blue, left y axis) and neonates born with microcephaly (red, right y axis) per epidemiological week in Bahia. Adapted from Ministry of Health of Brazil (2017). **(b)** 160 maternal sera (43 cases with microcephaly and 117 controls) were screened at 1:1,000 dilution for neutralization of ZIKV RVPs. Neutralization was expressed as the reciprocal of the luciferase activity normalized to no serum control. Samples below the dotted line (open symbols) were considered nonneutralizing. Each symbol represents the average of triplicate values for each donor. All triangles are maternal sera from microcephaly cases, and triangles pointing down represent ZIKV cases confirmed either by RT-PCR or IgM ELISA on cord blood. **(c)** ZIKV neutralization potency was determined using RVPs. The neutralization capacity was expressed as the reciprocal of the serum dilution resulting in 50% inhibition compared with RVPs alone (NT_50_). Each sample was evaluated in triplicate (*n* = 107, 40 cases with microcephaly and 67 controls). Three control samples that were identified as ZIKV neutralizers in the screening (panel b) were borderline in this assay and thus not plotted and omitted from further analysis. **(d)** IgG antibodies binding to ZEDIII were evaluated by ELISA. Binding was expressed as the reciprocal of the serum dilution resulting in 50% of maximal binding (BT_50_). Each value represents the average of two independent assays (*n* = 103, 40 cases with microcephaly and 63 controls). **(e)** Correlation between ZIKV neutralization potency, expressed as NT_50_, and ZEDIII binding, expressed as BT_50_. **(f)** Serum antibodies binding to the ZEDIII lateral ridge were determined by antigen competition ELISA and expressed as ΔBT_50_ (*n* = 103). **(g)** Correlation between ZIKV neutralization potency, expressed as NT_50_, and ZEDIII lateral ridge binding, expressed as ΔBT_50_. **(h)** IgG antibodies binding to UV-inactivated ZIKV were evaluated by ELISA. Optical densities were normalized by the control serum of a flavivirus naive individual vaccinated for YFV. Binding is expressed as the area under the curve (AUC) obtained in ELISA (*n* = 55, 27 microcephalies and 28 controls). **(i)** IgG antibodies binding to ZIKV NS1 protein were evaluated by ELISA. Optical densities were normalized as in panel h (*n* = 98, 39 cases with microcephaly and 59 controls). Each symbol represents an individual donor; black circles are from controls, and red triangles are from the microcephaly group. The P values in panels c, d, f, h, and i were determined with the Mann–Whitney test, and the mean and SD are shown. The P and ρ (rho) values for the correlation in panels e and g were determined with the Spearman test. *, P < 0.05; ***, P < 0.001. n.s., not significant.

Maternal sera were collected at the time of delivery from mothers in Salvador, Brazil, between November 2015 and February 2016 during the period of the microcephaly outbreak (Fig. 1 a). We obtained 160 samples for analysis: 43 from mothers of neonates with microcephaly and clinical and radiological evidence of CZS (see Materials and methods) and 117 from mothers of control newborns without microcephaly. Maternal sera were initially screened for ZIKV neutralization using luciferase-expressing reporter viral particles (RVPs) revealing a bimodal distribution (Fig. 1 b). Only women with higher reciprocal relative luciferase signal were considered to have been infected by ZIKV and studied further (*n* = 107, 40 microcephaly and 67 control cases; see Materials and methods).

To quantitate the ZIKV neutralizing activity, we determined the serum neutralization titer (NT_50_, Fig. 1 c; see Materials and methods). Serum neutralizing activity varied over two logs and was significantly higher in the microcephaly cases than in controls (P = 0.0004; Fig. 1 c). In ZIKV outbreak areas, serum neutralizing activity appears to correlate with levels of antibodies to the ZIKV envelope protein domain III (ZEDIII; Robbiani et al., 2017). To examine this relationship in our cohort, we evaluated serum IgG reactivity to ZEDIII by ELISA (50% binding titer [BT_50_]; Fig. 1 d). There was significantly higher binding to ZEDIII in the microcephaly group (P = 0.0003; Fig. 1 d). Moreover, ZEDIII antibodies correlated with ZIKV neutralization capacity in both microcephaly and control groups (Fig. 1 e). Antibodies to the lateral ridge of ZEDIII, which includes residues E393 and K394, are also associated with increased ZIKV neutralization (Robbiani et al., 2017). We analyzed lateral ridge antibodies in maternal sera by a newly developed antigen competition ELISA assay, in which antibodies are preincubated with either wild-type ZEDIII or with a ZEDIII mutant at E393/K394 before measuring residual IgG binding to ZEDIII (see Materials and methods). Similar to the antibodies to the whole ZEDIII, those to the ZEDIII lateral ridge displayed higher binding in the microcephaly group (P = 0.0425; Fig. 1 f) and were correlated with neutralization (Fig. 1 g). In contrast, antibody binding to UV-inactivated ZIKV was not significantly different between microcephaly and control groups, and the binding to ZIKV nonstructural protein 1 (NS1) was significantly reduced for microcephalies (Fig. 1, h and i). We conclude that serum neutralizing activity and ZEDIII antibodies correlate with each other and are significantly increased in women giving birth to microcephalic infants.

Dengue is endemic to the Salvador region in Brazil. To determine whether microcephaly is associated with the presence of antibodies to the EDIII of the four serotypes of DENV (DENV1–4) or to other flaviviruses (yellow fever virus [YFV] and West Nile virus [WNV]), we examined the quantity of IgG recognizing the EDIII of these viruses by ELISA (Robbiani et al., 2017). In contrast to the serologic reactivity to ZEDIII, IgG binding to the EDIII of all of the other flaviviruses tested was not significantly different between microcephaly and control groups (Table S1). Thus, we did not identify an association between ELISA detection of maternal antibodies to the EDIII of other flaviviruses and risk of microcephaly.

### Increased in vitro enhancement of ZIKV infection in mothers of microcephalic newborns

Antibody-dependent enhancement (ADE) of ZIKV infection by maternal serum was measured using luciferase-expressing ZIKV RVPs and the K-562 cell line. K-562 cells are resistant to infection by ZIKV but express the FcγRII receptor, which makes them susceptible to infection if the virus is bound by IgG (Halstead, 2003; Bardina et al., 2017). The luciferase signal (a surrogate of infection) was measured over a range of nine serum dilutions. Control experiments showed that the potent ZIKV neutralizing antibody Z004 neutralized the virus when present at high concentrations but enhanced infection of K-562 cells when diluted (Fig. S1, a and b). Enhancement was entirely dependent on Fc receptor binding, because it was abrogated by mutation of the Fc receptor binding site on Z004 (Z004-GRLR; Fig. S1 b). In contrast, neutralizing activity tested on permissive cells was independent of Fc receptor binding (Keeffe et al., 2018). Similar to the Z004 mAb, sera from nonpregnant ZIKV-infected individuals from Brazil and Mexico showed neutralizing activity at high concentrations and enhancing activity when diluted by this assay (Fig. S1 c; Robbiani et al., 2017). In contrast, samples obtained from flavivirus naive individuals showed neither neutralization nor enhancement (Fig. S1 c). Therefore, antibodies to ZIKV resemble antibodies to DENV in having concentration-dependent neutralizing and enhancing activity. The K-562 cell assay provides a means to characterize the combined impact (neutralization and enhancement) of the antibodies on ZIKV infection over a range of serum dilutions.

**Figure 2.**
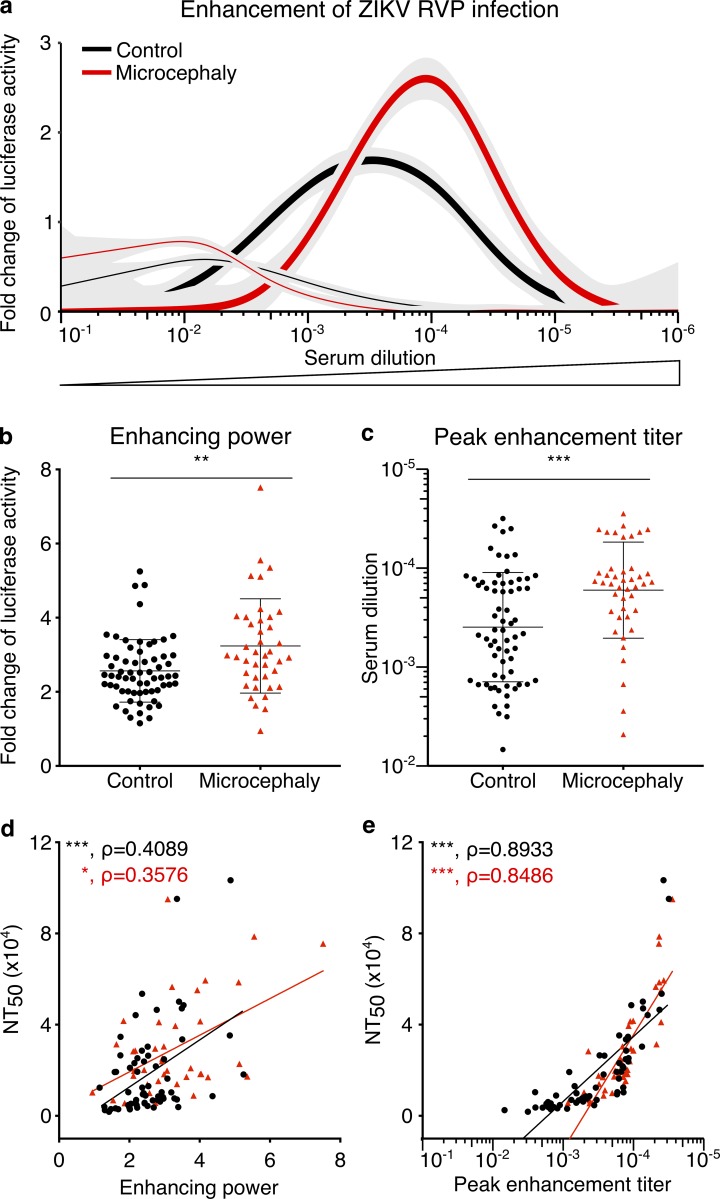
**Sera from mothers with microcephalic neonates have higher enhancing power and a higher peak enhancement titer. (a)** Enhancement of infection (fitted curves) by ZIKV RVPs is presented as the average of the fold change in luciferase activity of each group compared with control antibody (see Materials and methods). The thick lines represent control (*n* = 64) and microcephaly (*n* = 40) groups with ZIKV neutralizing activity, and thin lines represent samples that lack ZIKV neutralizing activity (empty symbols in Fig. 1 b). Sera were serially diluted and the enhancement of infection at each dilution for each group is shown. Standard errors are indicated in gray. The profile of the individual samples is shown in Fig. S2 a (*n* = 160). **(b)** Evaluation of the serum enhancing power. The enhancing power is defined as the fold increase of infection at peak enhancement titer for each serum sample (Fig. S1 a; Halstead, 2003). **(c)** Evaluation of the peak enhancement titer. The peak enhancement titer is the serum dilution at which maximum infection occurs for any tested sample (Fig. S1 a; Halstead, 2003). **(d)** Correlation between enhancing power and neutralization capacity, expressed as NT_50_. **(e)** Correlation between peak enhancement titer and NT_50_. The P values in panels b and c were determined with the Mann–Whitney test, and the mean and SD are shown. Symbols represent individual donors (*n* = 104). The P and ρ (rho) values for the correlations in panels d and e were determined with the Spearman test. *, P < 0.05; **, P < 0.01; ***, P < 0.001.

As expected, maternal sera with little or no neutralization activity (empty symbols in Fig. 1 b) displayed little or no enhancement (thin lines in Fig. 2 a; see also Fig. S2 a). In contrast, maternal sera with neutralizing activity showed varying degrees of enhancement (thick lines in Fig. 2 a; see also Fig. S2 a; Halstead, 2003). Sera from women delivering a microcephalic neonate showed a higher peak of luciferase expression (enhancing power, P = 0.0026; Figs. 2 b and S1 a) and higher peak enhancement titers (P = 0.0004; Figs. 2 c and S1 a; Halstead, 2003). Both features of the antibodies (the enhancing power and the peak enhancement titers) correlated positively with ZIKV neutralizing capacity (Fig. 2, d and e). We conclude that characteristics of the maternal antibodies associated with enhancement of infection in vitro differ between microcephaly and control groups.

### Clustering analysis reveals groups with distinct risk of microcephaly

**Figure 3.**
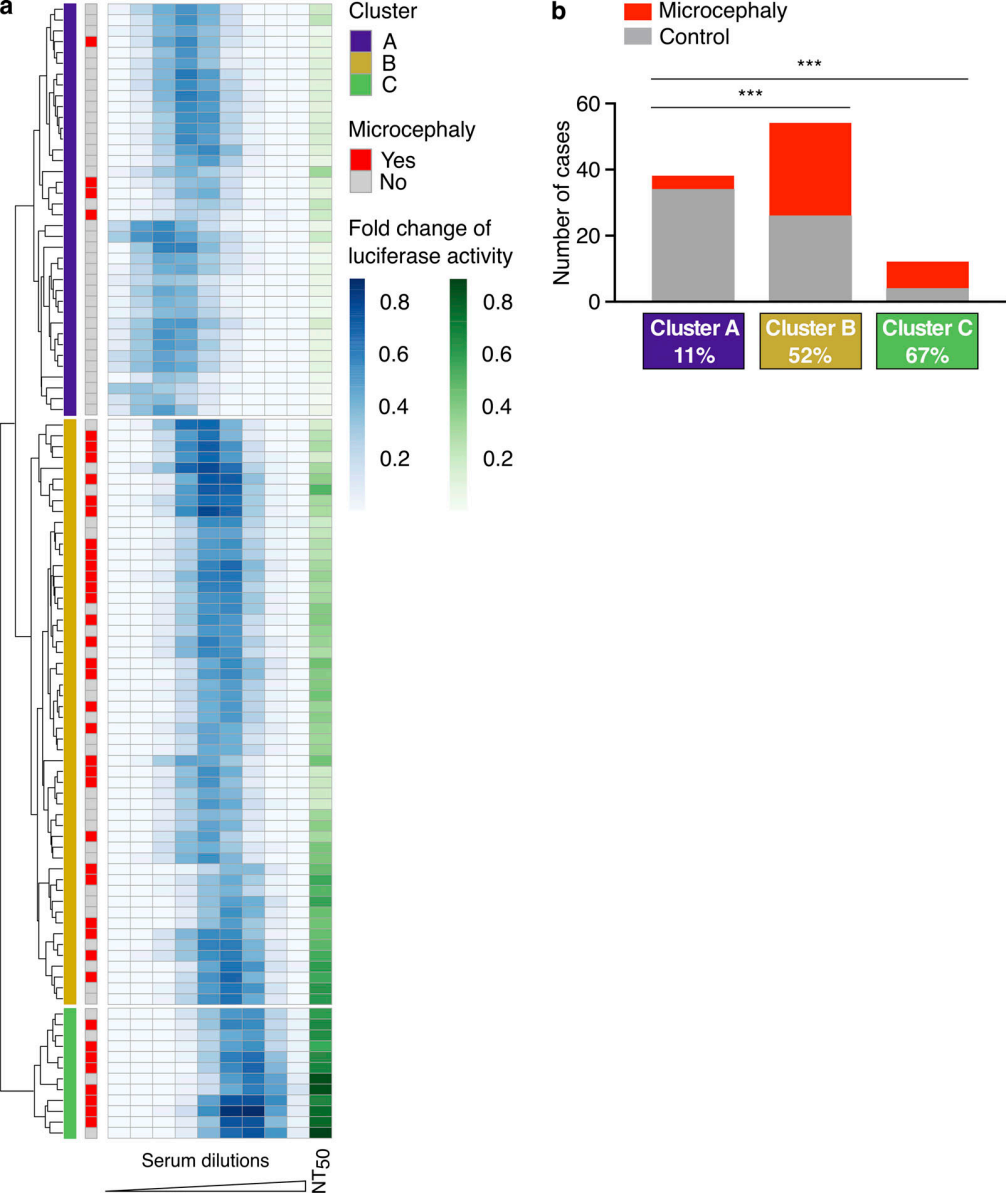
**Clustering analysis identifies groups with different retrospective risks of microcephaly in humans. (a)** Unsupervised hierarchical clustering of the log-normalized values for ZIKV RVP enhancement (ADE) combined with neutralization (NT_50_, *n* = 104). Clusters are indicated in the first column, and the presence or absence of neonatal microcephaly is indicated in the second column in red or gray, respectively. **(b)** Histogram with the number of microcephaly cases (red) and controls (gray) in the three clusters. Statistical analysis of the relative risks was performed using the Fisher’s exact test (***, P < 0.0001).

To determine whether a combination of serological features segregates individuals with increased risk of microcephaly, we performed unsupervised hierarchical clustering using the neutralization (NT_50_) and all serum dilution values from the ADE assay (Fig. 3 a). The samples segregated into three distinct clusters that differed in neutralization, enhancement profile, and relative risk of microcephaly (Fig. 3, a and b). Cluster A was characterized by enhancement at low serum dilution and low NT_50_ and correlated with the lowest rate of microcephaly (11%). Cluster B displayed intermediate levels of ZIKV enhancement of infection and neutralization and a 52% rate of microcephaly (P < 0.0001 vs. cluster A). Cluster C demonstrated enhancement at higher serum dilution, the highest NT_50_, and a 67% rate of microcephaly (P < 0.0001 and P = 0.35 vs. cluster A and B, respectively). Within each cluster, microcephaly and control samples were similar in enhancing power, peak enhancement titer, ZIKV neutralization, and ZEDIII binding (Fig. S3). Age of the mother and gestational age at birth were also similar within each cluster (Fig. S3).

Unsupervised hierarchical clustering was also performed using ADE values alone, enhancing power and peak enhancement titers, or by combining the ADE data with ZIKV neutralization. ADE values alone were sufficient to produce significant microcephaly associated clusters, which were improved by inclusion of NT_50_; other combinations did not (Figs. 3, S4, and S5). We conclude that antibody characteristics that are linked to enhancement of ZIKV infection in vitro are associated with significantly higher rates of microcephaly by ZIKV.

### ZIKV-infected nonhuman primate pregnancies

The exposure of the human cohort studied here to other flaviviruses is unknown, as is the timing of ZIKV exposure during pregnancy. Moreover, there is only limited clinical information on the control pregnancies. To address these limitations, we evaluated ZIKV neutralization and ADE of ZIKV infection in vitro as well as fetal outcome in macaques experimentally infected with ZIKV. Macaques are natural hosts for ZIKV (Vasilakis and Weaver, 2017), and although they do not develop ZIKV-associated microcephaly, infection during pregnancy is linked to neuropathology and fetal loss (Adams Waldorf et al., 2016, 2018; Coffey et al., 2018; Dudley et al., 2018; Martinot et al., 2018).

**Figure 4.**
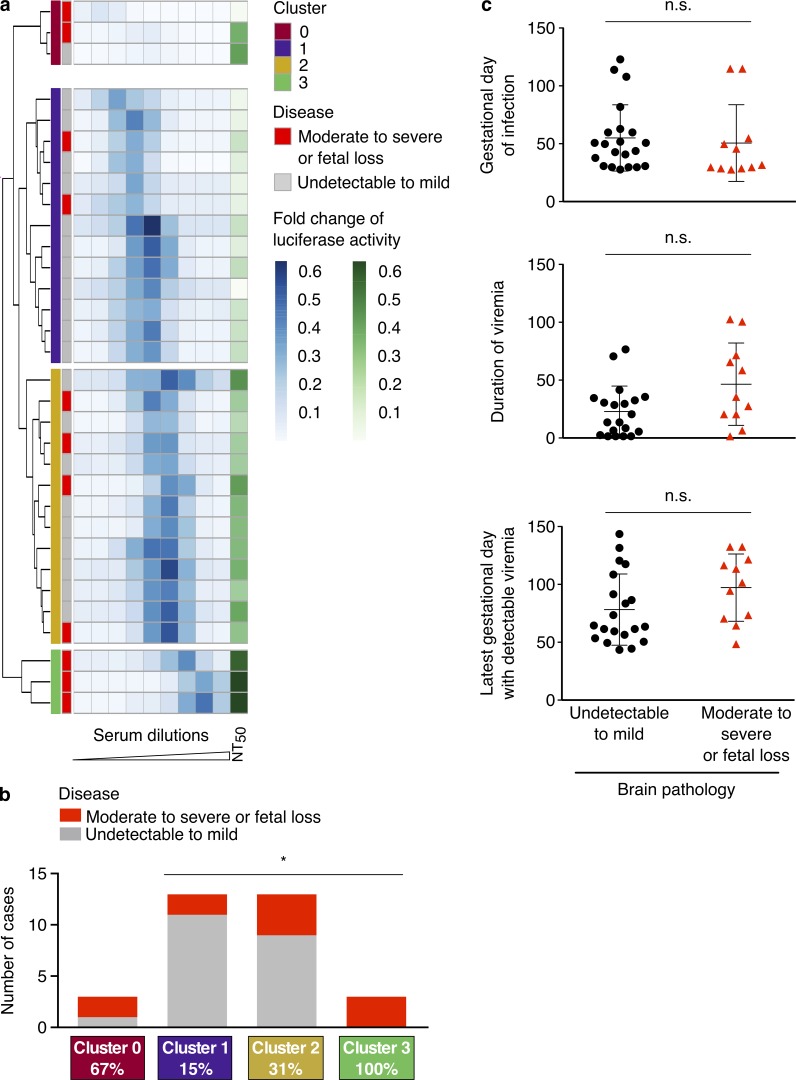
**Clustering analysis identifies groups with different retrospective likelihood of fetal brain damage in macaques. (a)** Unsupervised hierarchical clustering of the log-normalized values for ZIKV RVP enhancement (ADE) combined with neutralization (NT_50_, *n* = 32). Clusters are indicated in the first column and the degree of fetal brain damage is indicated in the second column in red or gray, respectively. **(b)** Histogram with the number of cases with moderate to severe fetal brain pathology or fetal loss (red) and controls with undetectable to mild lesion (gray) in the four clusters. The statistical analysis of the relative risks was performed using the Fisher’s exact test (*, P < 0.05). **(c)** Evaluation of gestational day (GD) of infection, duration of viremia, and last GD with detectable viremia. Duration of viremia is the difference between last detectable viremia and day of infection. The P values were not significant (P > 0.05 as determined with the Mann–Whitney test), and the mean and SD are shown. Symbols represent individual animals (*n* = 32, except in middle and bottom panel, where *n* = 31; see Table S2). n.s., not significant.

32 pregnant macaques were infected with ZIKV, their serum was collected at or near pregnancy termination, and fetal neuropathology was evaluated (see Materials and methods and Table S2). Serum neutralization (NT_50_) was determined using luciferase-expressing ZIKV RVPs, and enhancement was measured using the K-562 cell line as described above. To be consistent with the human samples, the data were subjected to the same unsupervised hierarchical clustering. Even though the statistical power is low given the small number of macaques, the analysis revealed four clusters, three of which closely mirrored the findings from the human cohort (Figs. 4 and S2 b). Cluster 1 (second from top in Fig. 4 a) displayed low NT_50_ and enhancement at low serum dilution, similar to cluster A (Fig. 3 a). Only 15% of macaque fetuses in this cluster showed brain injury (see Materials and methods). In contrast, clusters 2 and 3 resembled clusters B and C in humans (Fig. 3 a), with higher NT_50_ and enhancement at higher serum dilutions and higher rates of brain damage or early fetal loss compared with cluster 1 (31% and 100% respectively). A fourth cluster (cluster 0, top in Fig. 4 a) was characterized by antibody enhancement activity that was below the level of detection and high probability of adverse events (67%). There was no human equivalent to this group. Diseased and control samples were similar with respect to timing of infection and duration of viremia during pregnancy (Fig. 4 c). All macaques were flavivirus naive except one with evidence of WNV exposure (Table S2). We conclude that, similar to the results in humans, features of the ZIKV antibodies are associated with adverse fetal brain outcome.

## Discussion

In agreement with earlier reports, affected human pregnancies were associated with higher ZIKV neutralizing activity (Moreira-Soto et al., 2017; Netto et al., 2017). This observation is consistent with the finding that ZIKV viremia can persist for extended periods during pregnancy, leading to prolonged immune stimulation due to the continued exposure to viral antigens (Driggers et al., 2016; Meaney-Delman et al., 2016; van der Eijk et al., 2016). However, prolonged virus exposure should increase the antibody levels against all of the viral antigens. Instead, we find that antibodies that recognize distinct viral epitopes are differentially associated with microcephaly: ZEDIII antibodies are significantly increased, while antibodies directed against the UV-inactivated virus are not, and those to NS1 are significantly decreased.

Several factors have been proposed to influence fetal outcome during maternal ZIKV infection, including gestational age (Brasil et al., 2016; Kleber de Oliveira et al., 2016; Shapiro-Mendoza et al., 2017; Hoen et al., 2018), comorbidities (Aldo et al., 2016; Soares de Araújo et al., 2016), socioeconomic status (Netto et al., 2017), and genetic factors (Halai et al., 2017; Linden et al., 2017; Yuan et al., 2017; Caires-Júnior et al., 2018). Pre-exposure to structurally related flaviviruses like DENV can lead to antibodies that cross-react with ZIKV (Barba-Spaeth et al., 2016; Harrison, 2016; Stettler et al., 2016; Swanstrom et al., 2016; Heinz and Stiasny, 2017). These antibodies can cross-neutralize or cross-enhance ZIKV infection. As such, they have been suggested to facilitate vertical transmission through antibody-mediated transcytosis of ZIKV and by enhancing infection of FcR-expressing cells at the placental barrier (Quicke et al., 2016; Miner and Diamond, 2017; Zimmerman et al., 2018; Brown et al., 2019; Rathore et al., 2019). We found no significant differences in seroreactivity to flaviviruses other than ZIKV. Although our analysis was limited to ELISAs using the EDIII domains of DENV1–4, YFV, and WNV, our observations are consistent with published reports that pre-existing antibodies to DENV are not associated with increased risk of ZIKV-related microcephaly (Halai et al., 2017; Moreira-Soto et al., 2017; Pedroso et al., 2019). This result also agrees with the macaque experiments, which show that ZIKV injury to the fetal brain does not require prior flavivirus exposure. Although early infection and prolonged viremia are associated with congenital abnormalities (Driggers et al., 2016; Meaney-Delman et al., 2016; van der Eijk et al., 2016; Shapiro-Mendoza et al., 2017), neither is essential because brain pathology occurs despite late infection and without prolonged viremia (Fig. 4 c; Dudley et al., 2018).

The data show that features of the antibodies that modulate ZIKV infection in vitro are associated with a significantly altered risk of adverse outcome in vivo. Although sera from ZIKV-infected pregnant women showed antibody-enhancing activity in vitro regardless of fetal aberrations, sera from the microcephaly cases correlated with higher enhancing titers and power than nonmicrocephaly controls. Importantly, this observation was similar to what we observed in macaques: even though the low number of macaque samples weakens the power of the statistical analysis, in both primate species nonsupervised clustering revealed that ZIKV-related brain damage is increased in the clusters with higher neutralization and peak enhancement titers. While the data do not resolve causation versus correlation, they reveal a connection between features of the Zika antibodies and the likelihood of microcephaly. Our findings are consistent with the observation that antibodies that react with ZIKV enhance human placental macrophage infection in vitro and induce fetal loss in murine infection models (Zimmerman et al., 2018; Brown et al., 2019; Rathore et al., 2019).

In summary, the data reveal a correlation between antibody enhancement of ZIKV infection in vitro and pregnancy outcome for ZIKV infection in humans with validation in nonhuman primate models. Overall, the results support the hypothesis that an antibody-mediated mechanism may be associated with the pathogenesis of fetal brain injury and microcephaly, with significant implications for ZIKV vaccine development.

## Materials and methods

### Reagents

#### Antibodies

Z004 and Z004-GRLR were prepared by transient transfection of mammalian HEK-293-6E cells (Keeffe et al., 2018).

#### Viruses

ZIKV 2015 Puerto Rican PRVABC59 obtained from the Centers for Disease Control and Prevention was passaged and titrated as previously described (Lanciotti et al., 2016; Robbiani et al., 2017). This Puerto Rican strain of ZIKV is highly similar to the strains circulating in Brazil, and they all belong to the Asian/American lineage. The virus used on whole-virus ELISA was polyethylene glycol (PEG) precipitated with 40% PEG 6000 and UV inactivated for 30 min (with 9,999 μJ × 100 cm^2^ using a 254-nm UV lamp) on the same day of the harvest.

#### RVPs

Wild-type ZIKV RVPs used in neutralization and ADE assays were produced as previously reported (Robbiani et al., 2017).

#### Cells

K-562 human leukemia cells (ATCC CCL-243) for the enhancement assays were grown in RPMI with 10% FBS at 37°C under 5% CO_2_. Huh-7.5 human hepatocytes (Blight et al., 2002) for the neutralization assays were grown in DMEM supplemented with 1% nonessential amino acids and 5% FBS.

#### EDIII proteins

Wild-type and mutant ZEDIII proteins were expressed in *Escherichia coli*, refolded from inclusion bodies, and purified as described previously (Sapparapu et al., 2016; Robbiani et al., 2017). The expression plasmid encoding the ZEDIII protein mutated by alanine at the E393A/K394A residues was generated by QuikChange site-directed mutagenesis (Agilent Technologies) and confirmed by DNA sequencing. The primer used for mutagenesis was 5′-TTG​TCA​TAG​GAG​TCG​GGG​CGG​CGA​AGA​TCA​CCC​ACC​ACT​G-3′. DENV1, DENV2, DENV3, DENV4, YFV, and WNV EDIII proteins were produced in mammalian HEK293-6E cells as described previously (Robbiani et al., 2017).

### Clinical protocol and data and sample collection

Between November 2015 and February 2016, the study team identified neonates who were born at three hospitals (Hospital Geral Roberto Santos, Hospital Aliança, and Hospital Santo Amaro) in the city of Salvador, Bahia, Brazil, and requested written informed consent from the mothers to participate in the study. Neonates who had two or more head circumference measurements less than −2 SD with respect to the International Fetal and Newborn Growth Consortium for the 21st Century (INTERGROWTH-21st) sonographic reference (Villar et al., 2013) within 24 h of birth were defined as having microcephaly. Clinical and radiological evaluations, which included transfontanel ultrasound, cranial computed tomography, and examinations by a team of pediatric neurologists and ophthalmologists, were performed for infants with microcephaly to identify findings consistent with the CZS as defined by one or more of the following findings: characteristic neuroimaging findings as described by de Fatima Vasco Aragao et al. (2016); fetal brain disruption sequence such as overlapping cranial sutures and occipital skin fold; ophthalmological abnormalities such as macular scarring, chorioretinal atrophy, and other structural abnormalities (de Paula Freitas et al., 2016); axial or appendicular hypertonia; or congenital contractures such as talipes equinovarus or arthrogryposis. Sera from umbilical vein cord blood was obtained from infants for a ZIKV RT-PCR (Santiago et al., 2018) and for anti-ZIKV IgM testing at Instituto Evandro Chagas using an in-house IgM ELISA adapted from Martin et al. (2000). Maternal sera were obtained from peripheral venous blood samples collected within 2 d after delivery. Samples were selected for analysis from women with microcephalic neonates and clinical evidence of CZS, as well as from women with normocephalic neonates. Serum aliquots were shipped frozen, thawed at 4°C, heat inactivated at 56°C for 1 h, and stored at 4°C thereafter. The study protocol was approved by the Institutional Review Board committees of the Oswaldo Cruz Foundation (51889315.7.0000.0040), Hospital Geral Roberto Santos (no. 1.422.021), Yale University (HIC 1603017343), and The Rockefeller University (DRO-0898).

Biological materials are available where there are remaining samples (some samples have been depleted). In addition, for materials collected from human subjects, permission needs to be provided by the Oswaldo Cruz Foundation, Brazilian Ministry of Health due to rules and regulations of the Brazilian Ministry of Health and their ethical committees. Interested researchers can contact the corresponding authors to determine how such permissions can be obtained.

### Macaque protocols, sample collection, and analysis

Participating primate centers included the California National Primate Research Center (CNPRC), Washington National Primate Research Center (WaNPRC), Wisconsin National Primate Research Center (WNPRC), and Oregon National Primate Research Center (ONPRC). Cases were selected by investigators to include experimental ZIKV infections with pregnancies delivering or terminated experimentally after gestational day (GD) 125.

#### Care and use of nonhuman primates

All animals were either rhesus macaques (*Macaca mulatta*) or pigtailed macaques (*Macaca nemestrina*) and were cared for by staff at their respective primate centers, in accordance with the regulations and guidelines outlined in the Animal Welfare Act and the Guide for the Care and Use of Laboratory Animals. Work at CNPRC was approved by the Institutional Animal Care and Use Committee (IACUC) of University of California, Davis (#19695) and work at WaNPRC by the IACUC of the University of Washington (#4165-02). At WNPRC, work was approved by the University of Wisconsin-Madison College of Letters and Sciences and Vice Chancellor for Research and Graduate Education Centers IACUC, and the Wisconsin National Primate Research Center provided guidance and approval for the study protocol (G005401). At ONPRC, approval was by the IACUC (ONPRC protocol 1099), and the experiments were performed in strict accordance with the Guide for the Use of Laboratory Animals.

#### ZIKV infections

##### CNPRC

Two isolates were used: a 2015 Puerto Rico isolate (PRVABC59; GenBank accession no. KU501215; obtained from D. O’Connor, WNPRC) and a 2015 Brazil isolate (strain ZIKV/*H.sapiens*-tc/BRA/2015/Brazil_SPH2015; GenBank accession no. KU321639.1; obtained from Michael Busch, Vitalant Research Institute, San Francisco, CA). Aliquots were stored in liquid nitrogen and each time thawed right before the inoculation procedure. For each inoculation, the inoculum was adjusted to 10^3^ PFUs in 0.5 ml of RPMI-1640 medium and injected subcutaneously to simulate mosquito feeding. Each pregnant animal was inoculated three times at approximately GD30, 60, and 90 (corresponding to first and second trimester of human gestation; gestation of rhesus macaque is ∼165 d). The inoculations at GD30 and 90 consisted of PRVABC59, while the GD60 inoculation was performed with the Brazilian isolate.

##### WaNPRC

The following isolates were used: ZIKV strain isolated in Cambodia (FSS13025, 2010, GenBank accession no. KU955593) and ZIKV strain isolated in Fortaleza Brazil (Brazil 2015 [Fortaleza], GenBank accession no. KX811222). ZIKV was inoculated subcutaneously at five separate locations on the forearms, each with 10^7^ PFUs.

##### WNPRC

Two isolates of ZIKV were used: a 2013 French Polynesian isolate (ZIKV/*H.sapiens*-tc/FRA/2013/FrenchPolynesia-01_v1c1, GenBank accession no. KJ776791), originally isolated from a 51-yr-old female in France returning from French Polynesia with a single round of amplification on Vero cells, was obtained from Xavier de Lamballerie (European Virus Archive, Marseille, France), and a 2015 Puerto Rican isolate, PRVABC59 (ZIKV-PR; GenBank accession no. KU501215), originally isolated from a traveler to Puerto Rico with three rounds of amplification on Vero cells, was obtained from Brandy Russell (Centers for Disease Control and Prevention, Ft. Collins, CO). For each inoculation, the stock was thawed, diluted in PBS to 10^4^ PFUs/ml for each challenge and loaded into a 1-ml syringe that was kept on ice until challenge. 1 ml of inocula was administered subcutaneously over the cranial dorsum. At the conclusion of the procedure, animals were closely monitored by veterinary and animal care staff for adverse reactions and signs of disease.

##### ONPRC

ZIKV PRVABC59 was generously provided by the Centers for Disease Control and passed twice in C6/36 cells (ATCC) for the production of a working stock that was sequenced as previously described (Hirsch et al., 2017). Pregnant animals were inoculated once with 10^5^ focus-forming units subcutaneously in the arms.

#### Procedures

##### CNPRC

Animals in both the ZIKV-treated and placebo cohorts were sedated (using ketamine, 10 mg/kg intramuscular) at time zero (time of first virus inoculation; approximately GD30); at days 2, 3, 5, 7, 14, 21, and 30 (second ZIKV inoculation, GD60); at days 32, 37, 44, 51, and 60 (third ZIKV inoculation; GD90); and at days 62 and 67, and then weekly until time of euthanasia (between GD155 and 162) for sample collection and ultrasound monitoring. Blood samples were collected using venipuncture at every time point. Amniocentesis was conducted via ultrasound guidance starting at day 14 after inoculation (GD44) and then at all time points listed above with exception of days 32 and 62 after infection.

##### WaNPRC

The study design for animal inoculation, fetal magnetic resonance imaging, blood draw, delivery by cesarean section, and necropsy has been previously published (Adams Waldorf et al., 2016, 2018).

##### WNPRC

Animals were sedated (10 mg/kg intramuscular ketamine) for virus inoculation. Blood samples were acquired by venipuncture either while animals were sedated or in a squeeze cage daily from 1 to 10 d after infection and then twice a week while animals were viremic and once a week after that until animals delivered by cesarean section. Animals were sedated for ultrasound once per week. Ultrasound-guided amniocentesis was performed on some animals under sedation. Fetal loss was characterized by the absence of a fetal heartbeat during routine ultrasound examination or lack of fetal heartbeat at the time of birth. Fetectomy was performed the same day when fetal heartbeat was not detected during routine ultrasound.

##### ONPRC

Animals were sedated with ketamine for all procedures. Blood samples were collected using venipuncture. Ultrasounds were performed routinely to assess fetal development and placental function. No amniocentesis procedures were conducted on ONPRC animals (Dudley et al., 2018; Hirsch et al., 2018).

#### Necropsy and tissue collection and processing

##### CNPRC, WaNPRC, and ONPRC

At necropsy, fetal and maternal tissues were surgically removed. All necropsies were performed by a board-certified pathologist and two technicians. Hysterotomy was performed on the pregnant macaques under inhalation anesthesia, and the fetus, placenta, fetal membranes, umbilical cord, and amniotic fluid were collected for detailed tissue dissection after fetal euthanasia with an overdose of sodium pentobarbital (≥120 mg/kg). Shortly after, the mother was euthanized. Each tissue was grossly evaluated in situ, excised, and collected in 10% neutral buffered formalin and routinely paraffin embedded and processed for histology.

##### WNPRC

At ∼155 d gestation, fetal tissues were surgically removed at laparotomy. The entire conceptus within the gestational sac was collected and submitted for necropsy. The fetus was euthanized with an overdose of sodium pentobarbital (50 mg/kg). Tissues were fixed in 10% neutral buffered formalin for 14 d and transferred into 70% ethanol until routinely processed and embedded in paraffin.

#### Histology of fetal brain

##### CNPRC, WaNPRC, WNPRC, and ONPRC

Fetal brain (13–20 sections/animal) was evaluated blindly by pathologists and assigned, based on severity, to either no detectable to mild disease group, or moderate to severe or fetal loss group. Slides were initially evaluated at the participating centers and then sent to CNPRC for an additional blind review.

#### WNV ELISA

##### CNPRC and WNPRC

Screening of macaque plasma IgG binding to WNV was performed with simian WNV ELISA plates as instructed by the manufacturer (XpressBio).

##### ONPRC

High protein-binding ELISA plates (Costar) were coated overnight at 4°C with WNV particles diluted in PBS. The plates were blocked with PBS containing 2% milk and 0.05% Tween-20 for 1 h at room temperature. Plates were washed with ELISA wash (PBS containing 0.05% Tween-20). Twofold dilutions of plasma samples were added to the plates and incubated for 2 h. Plates were washed and incubated with diluted secondary anti-monkey IgG/IgA/IgM conjugated to HRP (Rockland) for 30 min. Plates were washed and detected using ortho-phenylenediamine substrate followed by the addition of HCl. Plates were read using a Synergy HTX Microplate Reader (BioTek) at 490 nm.

### Neutralization and ADE assays

Neutralization of luciferase-encoding RVPs by maternal sera using the ZIKV RVPs was performed as previously described (Robbiani et al., 2017). The screening for neutralization was with all 160 samples, diluted at 1:1,000 with BA-1 medium (Medium 199 [H7653; Sigma], 1% BSA, 1,400 mg/liter sodium bicarbonate, and 100 U/ml Pen/Strep). Due to the bimodal structure of the reciprocal relative luciferase signal, a Gaussian mixture model was fitted to the data, and two biologically meaningful groups were identified (threshold = 1.95, dotted line in Fig. 1 b): ZIKV neutralizers (*n* = 107) and nonneutralizers (*n* = 53). The group assignment and threshold calculation were performed using the R package mclust. Among 43 of the maternal sera from microcephaly cases, 40 (93%) were above the 1.95 threshold, whereas among 117 of the controls, 67 (57%) were above. Furthermore, among maternal sera from 23 microcephaly cases who had anti-ZIKV IgM in the cord blood or positive ZIKV RT-PCR results (Oliveira-Filho et al., 2018), 21 (91%) were above the 1.95 threshold. We therefore defined mothers that scored >1.95 in the screening as having been exposed to ZIKV, and their samples were studied further.

To quantitate the neutralization capacity, the human or macaque serum was serially diluted 1:3 in BA-1 medium (1:900 to 1:1,968,300, eight serum dilutions in total), and the reciprocal of the serum dilution that resulted in 50% inhibition compared with RVP alone was reported as the 50% neutralization titer (NT_50_).

The ADE assay was similar to the neutralization assays with RVPs, except that Fc-receptor–bearing K-562 cells were used, and the cells were in 96-well plates coated with 0.01% poly-L-lysine (Sigma; Littaua et al., 1990). For ADE, the serum was serially diluted 1:3 in BA-1 medium (1:50 to 1:328,050, nine dilutions in total). The luciferase signal was normalized to the signal obtained with 10 ng/ml of antibody Z004, which was present on the same plate, and expressed as the fold change in luciferase activity. Profile curves were fitted to ADE data using the generalized additive model function from the mgcv R package with the formula y ~ ns(x, df = 7).

### ELISA assays

#### Whole-virus ELISA

The amount of serum IgG binding to whole, UV-inactivated ZIKV was measured by ELISA. ELISA plates were coated with 5 µg/ml of mouse anti-flavivirus 4G2 antibody in carbonate-bicarbonate buffer (Sigma) and incubated for 2 h at 37°C. Between each step, the plates were washed three times with PBS-T (PBS with 0.05% Tween-20). Blocking was with 1% BSA and 0.1 mM EDTA in PBS-T for 1 h at 37°C. PEG-precipitated and UV-inactivated ZIKV was diluted 1:3 in PBS, and the equivalent of 4 × 10^3^ PFUs was added to each well and incubated overnight at 37°C. Sera were serially diluted 1:10 in PBS-T (1:100 to 1:100,000, four dilutions in total) and incubated for 2 h at 37°C. Secondary HRP-conjugated goat anti-human IgG (0.16 µg/ml; Jackson ImmunoResearch) was added for 1 h at 37°C. Plates were developed using ABTS substrate (Life Technologies) and read at 405 nm. The area under the curve was computed using natural spline interpolation with the Miscellaneous Esoteric Statistical Scripts package from R. Whole-virus ELISA was performed on 55 samples due to limiting amounts of virus.

#### ZEDIII ELISA and antigen competition ELISA

ELISA assays were used to evaluate the amount of serum IgG binding to whole ZEDIII (BT_50_) or to the ZEDIII lateral ridge by antigen competition (ΔBT_50_). Serial dilutions of maternal sera were incubated overnight at 4°C, nutating, in V-bottom 96-well plates in the presence of buffer only (to determine the BT_50_) or in the presence of saturating concentrations of either wild-type ZEDIII or ZEDIII_E393A/K394A_ (to determine the ΔBT_50_). The saturating concentration of ZEDIII protein was previously determined as 10 µg/ml. After overnight incubation, the samples were added to ELISA plates to measure the residual serum IgG antibodies binding to ZEDIII as previously described (Robbiani et al., 2017), with the exception that the signal was enhanced by two amplification steps. First, after incubating with goat anti-human IgG-HRP (catalog number 109–035-098; 1 h, room temperature; Jackson ImmunoResearch) and washing with PBS containing Tween-20 0.05%, anti-goat IgG-biotin was added (catalog number 705–065-147; 1 h, room temperature; Jackson ImmunoResearch). Second, after washing, streptavidin-HRP was added (catalog number 016–030-084; 1 h, room temperature; Jackson ImmunoResearch). After the final washes, the reaction was developed with ABTS substrate and read at 405 nm. The amount of serum IgG binding to whole ZEDIII was determined by nonlinear regression analysis, and the reciprocal of the serum dilution that resulted in 50% of maximal binding is reported as the BT_50_. The amount of ZEDIII lateral ridge–specific antibodies was determined by the difference of the BT_50_ values measured after blocking with ZEDIII or ZEDIII_E393A/K394A_ (ΔBT_50_).

#### Flavivirus EDIII ELISA

ELISA to a panel of flavivirus EDIII proteins was performed as described previously (Robbiani et al., 2017) in two independent experiments. The optical density for each sample was normalized to a flavivirus naive control serum before averaging the reading of both experiments.

#### ZIKV NS1 ELISA

Serum IgG binding to ZIKV NS1 protein was detected by ELISA as described previously (Magnani et al., 2017), with some alterations. Briefly, ZIKV NS1 protein (catalog number MBS568704; MyBioSource) was incubated overnight at room temperature, the blocking solution was the same as used for the whole virus ELISA described above, serum samples were diluted 1:200, and goat anti-human IgG HRP (Jackson ImmunoResearch) was diluted at 1:5,000 in blocking solution.

### Statistics and clustering analysis

Statistical analysis was with Prism 8 software (including for Pearson’s r) and two tailed. Box-plot elements in Fig. S3 b are defined as follows: center line, median; box limits, upper and lower quartiles; and whiskers, 1.5× interquartile range. ADE, neutralization (NT_50_), ZEDIII binding (BT_50_), enhancing power, and peak enhancement titer values were used in unsupervised hierarchical clustering analysis using R language. Except for the analysis in Fig. S5 b, data were rescaled to ADE values and log transformed before clustering analysis using Euclidean distance and UPGMA (unweighted pair group method with arithmetic mean) agglomeration method for *dist* and *hclust* R functions, respectively. A heatmap and UPGMA tree were created using the *Pretty Heatmaps* (*pheatmap*) R package. The optimal number of clusters per dataset was automatically chosen using the *NbClust* package, which provides 30 indices for determining the number of clusters and proposes the best clustering scheme from the different results obtained by varying all combinations of cluster numbers, distance measures, and clustering methods.

### Online supplemental material

Fig. S1 demonstrates the ADE of ZIKV RVP infection by recombinant antibodies and sera. Fig. S2 shows the individual profile of human and macaque maternal sera in the in vitro ADE assay. Fig. S3 compares the serological and clinical parameters of control and microcephaly groups in each cluster of Fig. 3. Fig. S4 demonstrates the unsupervised hierarchical clustering analysis of ADE and NT_50_ values alongside the IgG ELISA values. Fig. S5 demonstrates the unsupervised hierarchical clustering analysis using ADE alone, peak enhancement titer with enhancing power, or ADE in combination with NT_50_ and BT_50_ values. Table S1 shows the IgG reactivity of maternal sera to the EDIII of a panel of flaviviruses. Table S2 provides information on the macaques in this study.

## Supplementary Material

Supplemental Materials (PDF)

Table S1 (Excel file)

Table S2 (Excel file)
