# Supplementary material for: Risk of Zika microcephaly correlates with features of maternal antibodies
Source: J Exp Med. 2019 Aug 14;216(10):2302–15. doi: 10.1084/jem.20191061 (PMC6781003; doi:10.1084/jem.20191061)
Supplement: Supplemental Materials (PDF) [file JEM_20191061_sm.pdf]

Supplemental material

Robbiani et al., https://doi.org/10.1084/jem.20191061

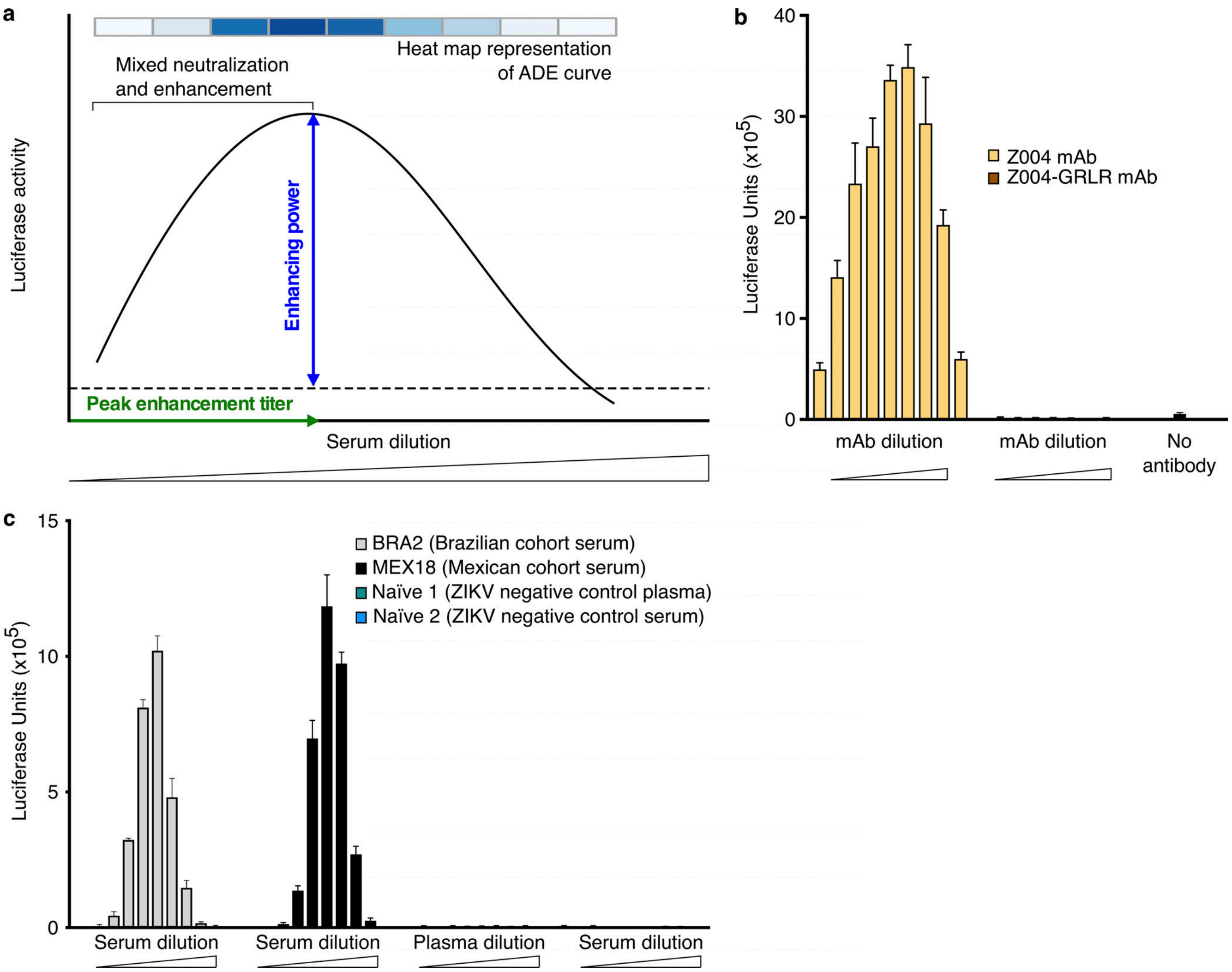

Figure S1. **ADE of ZIKV RVP infection. (a)** Cartoon diagram defining the parameters of ADE: enhancing power (in blue) and peak enhancement titer (in green). Adapted from Halstead, 2003. Heat map representation is shown on top. **(b)** Control ADE assays using ZIKV RVPs. The neutralizing mAb Z004, and its Fc mutant, Z004-GRLR, with impaired binding to FcγR (Horton et al., 2008) were evaluated for ADE at 1:3 serial dilutions starting at 10 µg/ml. Triangles represent serial antibody dilutions, from left to right. **(c)** ZIKV neutralizing sera from Brazil and Mexico (Robbiani et al., 2017) and samples from flavivirus naive individuals from New York City were evaluated for ADE at 1:3 serial dilutions starting at 1:50. Error bars are SD.

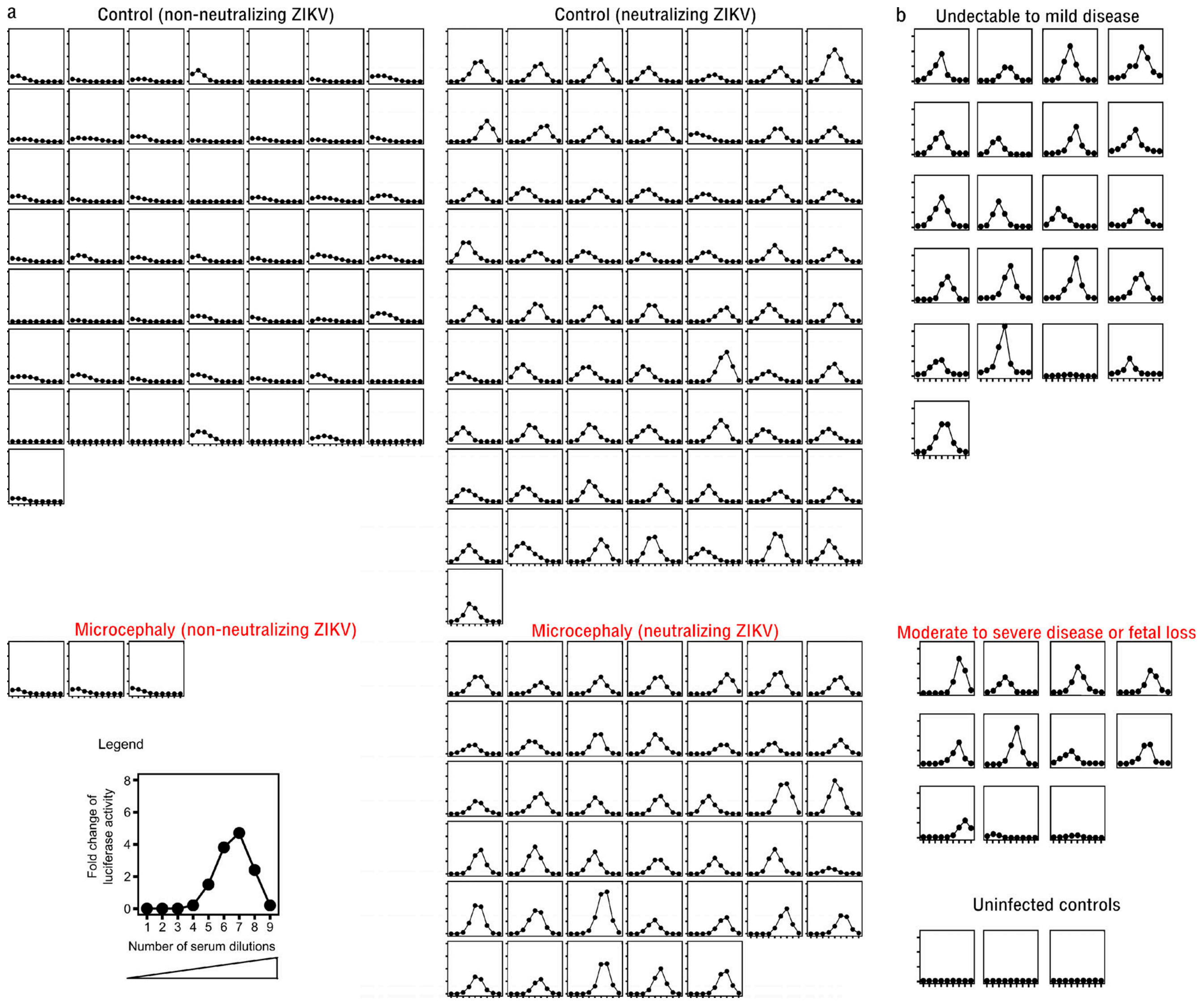

Figure S2. **Enhancement of ZIKV RVPs infection by individual maternal sera. (a)** Enhancement profiles of individual human maternal sera from the microcephaly group (red thick line in Fig. 2 a), control group (black thick line in Fig. 2 a), and individuals without ZIKV neutralizing activity (thin lines in Fig. 2 a, corresponding to empty circles and triangles in Fig. 1 b). **(b)** Enhancement profiles of individual macaque maternal sera from the disease group (red in Fig. 4), control group (gray in Fig. 4), and uninfected macaques (not shown in Fig. 4).

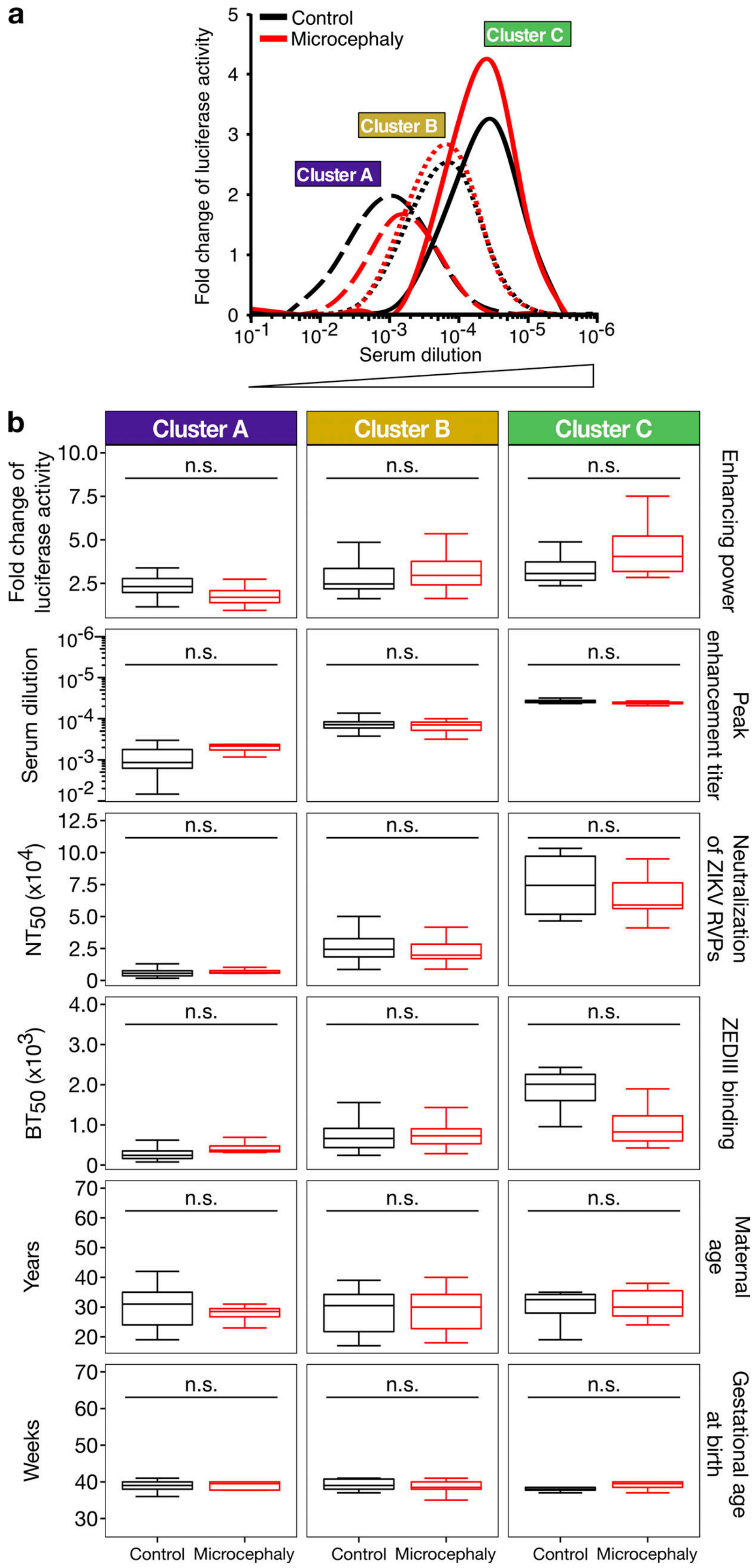

Figure S3. **Analysis of the individual serological and clinical parameters in clusters A, B, and C. (a)** ADE profile of ZIKV RVPs infection (fitted curves) from the three distinct clusters (see Figs. 2 a and 3 a). Dashed lines represent cluster A, dotted lines cluster B, and filled lines cluster C. **(b)** Enhancing power, peak enhancement titer, neutralization of ZIKV RVPs, ZEDIII binding, maternal age, and gestational age at birth in the control and microcephaly groups were evaluated within each cluster. The P values were not significant (P > 0.05 as determined with the Mann–Whitney test), and the mean and SD are shown.

JEM

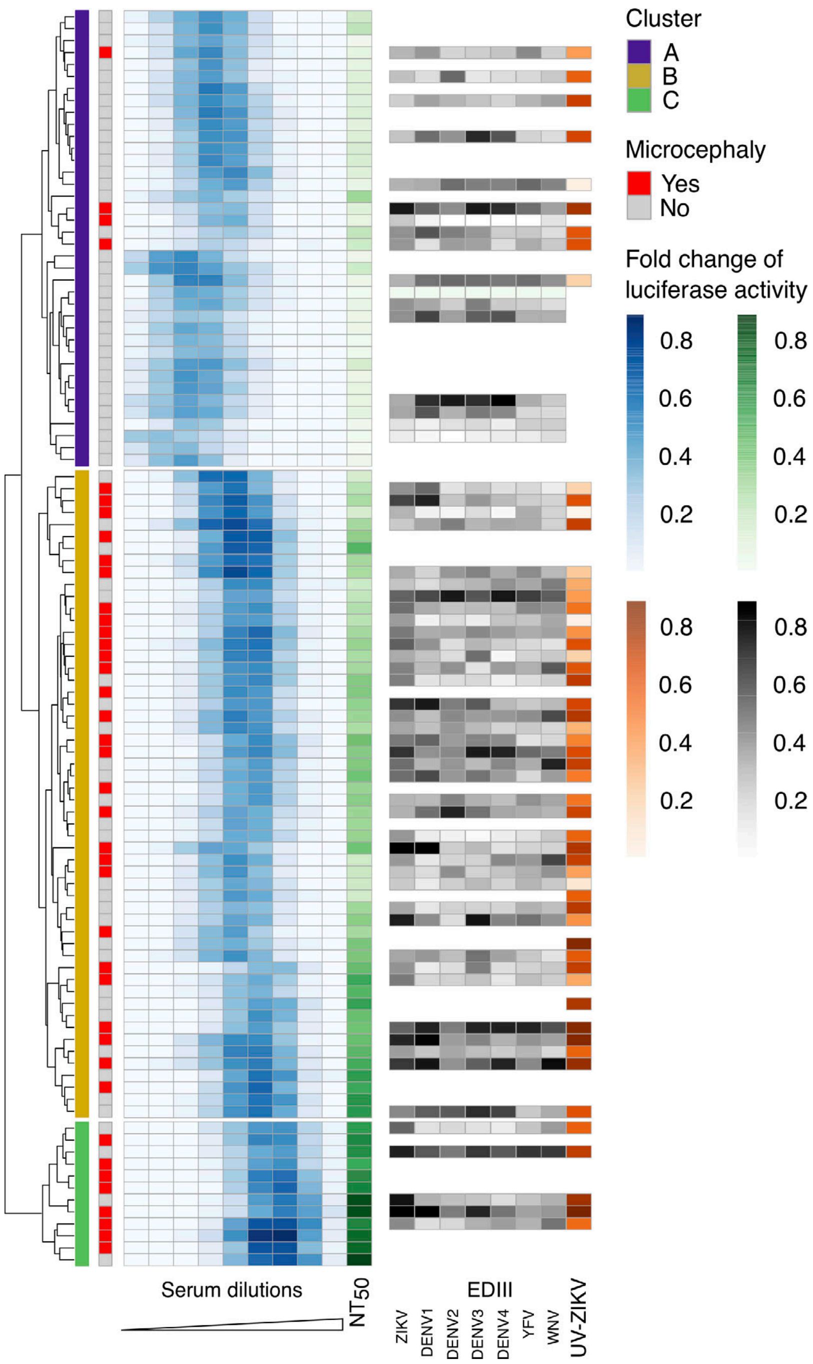

Figure S4. **Clustering analysis combined with IgG binding values.** The unsupervised hierarchical clustering analysis was performed as in Fig. 3 a using the values from the ADE and neutralization experiments. Shown on the right are the log-normalized values for IgG binding to the EDIII of a panel of flaviviruses (grayscale, related to Table S1) or to UV-inactivated ZIKV (orange scale, related to Fig. 1 h), as determined by ELISA.

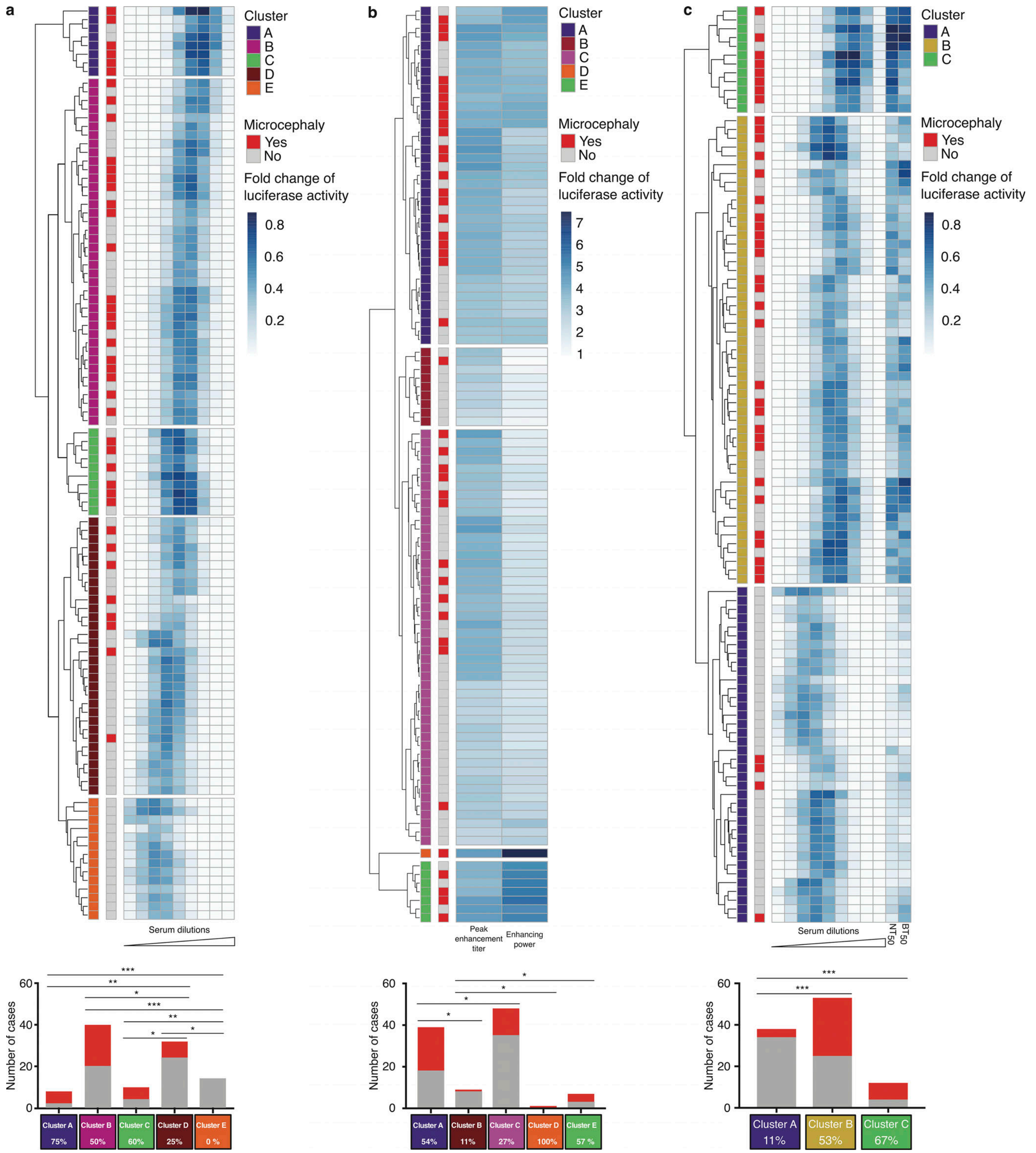

Figure S5. **Unsupervised hierarchical clustering analyses with distinct parameters. (a)** The unsupervised hierarchical clustering analysis with heat map of the log normalized values for ZIKV enhancement only (ADE, *n* = 104). **(b)** Same as in panel a but with peak enhancement titer and enhancing power (values not log normalized, *n* = 104). **(c)** Same as in panel a but for the log-normalized values of ZIKV enhancement (ADE), ZIKV neutralization (NT_{50}), and ZEDIII binding (BT_{50}) of each maternal serum (*n* = 103). In all panels, different clusters are indicated in the first column and presence or absence of microcephaly are indicated in the second column in red or gray, respectively. Histograms represent the number of microcephalies (red) and controls (gray) in each cluster. The percentage of microcephalies in each cluster is shown at the bottom. The statistical analysis of the relative risks was performed using the Fisher's exact test (*, $P < 0.05$; **, $P < 0.01$; ***, $P < 0.001$). n.s., not significant.

https://doi.org/10.1084/jem.20191061

Tables S1 and S2 are provided as Excel files online. Table S1 shows the IgG reactivity of maternal sera to the EDIII of ZIKV, dengue (serotypes 1–4), yellow fever, and WNV. Table S2 presents information on the macaques used in this study.

References

Halstead, S.B. 2003. Neutralization and antibody-dependent enhancement of dengue viruses. *Adv. Virus Res.* 60:421–467. https://doi.org/10.1016/S0065-3527(03)60011-4

Horton, H.M., M.J. Bernett, E. Pong, M. Peipp, S. Karki, S.Y. Chu, J.O. Richards, I. Vostiar, P.F. Joyce, R. Repp, et al. 2008. Potent in vitro and in vivo activity of an Fc-engineered anti-CD19 monoclonal antibody against lymphoma and leukemia. *Cancer Res.* 68:8049–8057. https://doi.org/10.1158/0008-5472.CAN-08-2268

Robbiani, D.F., L. Bozzacco, J.R. Keeffe, R. Khouri, P.C. Olsen, A. Gazumyan, D. Schaefer-Babajew, S. Avila-Rios, L. Nogueira, R. Patel, et al. 2017. Recurrent Potent Human Neutralizing Antibodies to Zika Virus in Brazil and Mexico. *Cell.* 169:597–609.e11. https://doi.org/10.1016/j.cell.2017.04.024
